# Novel Hierarchical Fall Detection Algorithm Using a Multiphase Fall Model

**DOI:** 10.3390/s17020307

**Published:** 2017-02-08

**Authors:** Chia-Yeh Hsieh, Kai-Chun Liu, Chih-Ning Huang, Woei-Chyn Chu, Chia-Tai Chan

**Affiliations:** Department of Biomedical Engineering, National Yang-Ming University, Taipei 112, Taiwan; kerrhsieh@ym.edu.tw (C.-Y.H.); g30104026@ym.edu.tw (K.-C.L.); g39604001@ym.edu.tw (C.-N.H.); wchu@ym.edu.tw (W.-C.C.)

**Keywords:** fall detection algorithm, multiphase fall model, wearable sensor

## Abstract

Falls are the primary cause of accidents for the elderly in the living environment. Reducing hazards in the living environment and performing exercises for training balance and muscles are the common strategies for fall prevention. However, falls cannot be avoided completely; fall detection provides an alarm that can decrease injuries or death caused by the lack of rescue. The automatic fall detection system has opportunities to provide real-time emergency alarms for improving the safety and quality of home healthcare services. Two common technical challenges are also tackled in order to provide a reliable fall detection algorithm, including variability and ambiguity. We propose a novel hierarchical fall detection algorithm involving threshold-based and knowledge-based approaches to detect a fall event. The threshold-based approach efficiently supports the detection and identification of fall events from continuous sensor data. A multiphase fall model is utilized, including free fall, impact, and rest phases for the knowledge-based approach, which identifies fall events and has the potential to deal with the aforementioned technical challenges of a fall detection system. Seven kinds of falls and seven types of daily activities arranged in an experiment are used to explore the performance of the proposed fall detection algorithm. The overall performances of the sensitivity, specificity, precision, and accuracy using a knowledge-based algorithm are 99.79%, 98.74%, 99.05% and 99.33%, respectively. The results show that the proposed novel hierarchical fall detection algorithm can cope with the variability and ambiguity of the technical challenges and fulfill the reliability, adaptability, and flexibility requirements of an automatic fall detection system with respect to the individual differences.

## 1. Introduction

According to the International Database of the U.S. Census Bureau, the average percentage of individuals over 65 years old was 17% of the population in more developed countries, such as the United States, Japan, Germany, etc., in 2015, and this percentage is expected to reach 30% in 2050. Population aging is a common phenomenon in many countries, particular developing and developed countries, due to declining fertility rates and increasing life expectancy [1]. Falls among the elderly are public health issues and threats which can implicate quality of life and cause severe disabilities that impact independent living [2,3]. Fall-related injuries among the fallers found helpless in their homes are more serious than those among people who are helped within 12 h [4]. The association between the mortality rates and waiting time for rescue shows a positive correlation [4,5]. In addition to causing disability or functional impairment, non-fatal injurious falls also have psychological and social effects [6]. Fear of falling again makes the elderly lose self-confidence in their ambulant safety, limiting the function of activities of daily living (ADLs) [7].

In order to improve the quality of home healthcare services, two kinds of strategies are proposed to deal with the occurrence of falls, including fall prevention and fall detection [8]. The fall prevention strategy analyzes risk factors, and then delivers targeted interventions to reduce the incidence of falls [9,10]. The environmental factors include obstacles, poor light, loose carpets, lack of safety equipment, and weather; the most frequently mentioned physiological reasons for falls are losing balance, a history of falls, functional and cognitive impairments, medication use, postural blood pressure, muscle weakness, and visual impairment [7,11]. Existing ways to prevent falls from occurring include muscular strength and body balance training, and a preventative checklist can be created to minimize the risk of falls in inimical environments. Unfortunately, falls cannot be completely prevented, so a fall detection system is essential for the elderly.

A fall detection system is considered an assistive system aiming to send an alert when a fall event happens. There are two types of fall detection systems, including user-manual and automatic systems. User-manual fall detection systems are designed to send emergency messages by user manipulation. However, such systems cannot provide first aid for the faller during loss of consciousness. Alternatively, automatic fall detection systems are proposed to detect falls without any user manipulation when the faller loses consciousness. Automatic fall detection systems can be classified into two main categories according to their sensor type: one is an ambient device-based fall detection system, while the other is a wearable-based fall detection system [12,13]. Ambient devices are arranged in the smart environment, and they include cameras [14], infrared sensors, acoustic sensors, vibration sensors, and pressure sensors [15]. These ambient devices have good performance in confined environments, such as living rooms, bathrooms, or laboratories. However, these devices are not feasible in uncontrolled environments. Wearable sensor–based fall detection systems, by contrast, can detect falling at any time, anywhere, while the user wears the sensor(s). Lin et al. presented a wearable micro-mercury switch integrated with an optical sensor to enhance the fall detection rate of the wearable device [16]. Chen et al. used a tri-axial accelerometer attached to the waist to detect falls and a network of fixed motes to locate the victim [17]. Bourke and Lyons used only one bi-axial gyroscope mounted on the trunk to detect falls [18]. In this work, the proposed fall detection algorithm aims to automatically detect a critical fall, where fall is defined as “an event in which a person inadvertently comes to rest on the ground, floor, or lower level as a consequence of the following: sustaining a violent blow, loss of consciousness, sudden onset of paralysis, as in a stroke or an epileptic seizure”, given the person cannot call for help by himself/herself [11,19].

A fall detection algorithm plays an important role in an automatic fall detection system. There are two common technical issues and challenges that should be tackled in order to provide a reliable fall detection algorithm. The first is variability, as falls can happen involuntarily and suddenly in various forms and directions in daily living. Falls might happen when you are walking, standing, and they frequently happen in activity transitions, such as during bed and chair exits [20]. The second is ambiguity, as some features of falls are similar to the features of ADLs, which might confuse the fall detection system. For example, serious falling events can occur with strong impact and energy, which are similar to jumping or running in daily living. Additionally, light falling events occur with a smaller impact and energy than those of serious falling events, but the light falling event might be similar to lying down in daily living. These technical issues and challenges may lead to most fall detection algorithms being insufficient for automatic fall detection systems when only the specific event is considered, such as impact, posture after a fall, and change of velocity during a fall. Several well-known studies have proposed a multiphase fall model to provide more fine-grained observation of the fall event for automatic fall detection systems, where the fall can be divided into different phases, such as four phases (the pre-fall, critical, post-fall, and recovery phases) [21], and five phases (the pre-fall, falling, impact, resting, and recovery phases) [22]. For instance, the post-fall and pre-fall activity might highly influence the impact signal. Therefore, a multiphase fall model has the potential to deal with the aforementioned technical challenges and to provide a more fine-grained level of information for a fall detection system.

This study aims to accurately detect falls during the execution of activities of daily living using wearable sensors. We propose a novel hierarchical fall detection algorithm including threshold-based and knowledge-based approaches to detect a fall event. Firstly, the sets of the thresholds are determined to identify the absolute falls and ADLs in a threshold-based approach. The advantages of a threshold-based approach are the low computing complexity and ease of implementation. However, it is difficult to set an appropriate threshold value since there is an overlap of peak acceleration values generated by falls and ADLs. Therefore, the ambiguous problem is solved by the knowledge-based approach. Relying on a multiphase fall model, including free fall, impact, and rest phases, knowledge-based approaches can improve the performance matrices of the simple threshold-based fall detection algorithms.

The rest of this work is organized as follows: In Section 2, we briefly expand on the related work of fall detection, such as threshold-based and machine learning-based fall detection algorithms. The proposed hierarchical fall detection algorithm, including threshold-based and knowledge-based approaches, and the performance evaluation are introduced in Section 3. The results analysis and discussion are described in detail in Section 4. Finally, we summarize the conclusions of the proposed hierarchical fall detection algorithm in Section 5.

## 2. Related Work

Fall detection systems using wearable sensors are still an open research area that involves a sequence of signal processing, pattern recognition, and machine learning techniques. Generally, the framework of automatic fall detection systems is sensor data collection, a fall detection algorithm, and an emergency alarm [23]. This work is dedicated to developing a fall detection algorithm; therefore, this section especially focuses on reviewing important topics of existing fall detection algorithms.

### 2.1. Fall Detection Algorithm

Sensor placement is a critical issue for the development of wearable sensor–based fall detection algorithms. The most common wearing positions are the waist, wrist, trunk, thigh, back, ankle, foot, neck, and head [23]. The waist and trunk are near the center of mass of the human body and the neck will maintain the head’s balance when humans perform ADLs, so the sensor attached to the waist, trunk, or head can detect larger accelerations when the body hits the ground.

Fall detection algorithms of wearable sensor–based fall detection systems are primarily classified into two parts: threshold-based algorithms and machine learning-based fall detection algorithms [12,24]. Some studies have aimed at evaluating the effectiveness of different wearing positions and detection algorithms, as shown in Table 1.

#### 2.2.1. Threshold-Based Fall Detection Algorithms

The threshold-based fall detection methods discriminate between falls and ADLs when the peak values are below or above the threshold [25,26]. The advantages of threshold-based techniques are the low computing complexity and easy implementation in wearable sensors. However, threshold-based techniques are not suitable to detect different types of falls, because thresholds are designed according to the body’s experience during falling and fixed thresholds cannot fulfill all kinds of individual habits of activities in daily living.

Bourke et al. [25] used a single threshold, which is an upper fall threshold related to the peak impact force during falling, or a lower fall threshold related to the acceleration before contact with the ground, to detect falls by three accelerometer sensors mounted on the trunk and thigh. The results showed that the 3.52*g* (1*g* = 9.81 m/s^2^) upper fall threshold for the trunk had the highest specificity, and suggested that the trunk was the optimal wearing position for a fall sensor. Kangas et al. [27] used a single threshold–based fall detection algorithm with posture detection after the fall with a tri-axial accelerometer attached at the waist, wrist, or head to investigate the placement of a fall detection sensor. The results showed that the head-worn accelerometer provided perfect results, and the authors suggested that the head was a reasonable wearing position for fall detection. Designing more complex algorithms than the single threshold–based fall detection algorithms, Kangas et al. [28] evaluated different low-complexity fall detection algorithms using accelerometers attached at the waist, wrist, and head. The results ultimately indicated that the effective sensor placements were the waist and head. The sensor at the head level had the highest accuracy, but the usability and user’s acceptance should be considered in more detail, i.e., ergonomics. In conclusion, an accelerometer worn on the waist might be an optimal choice for a wearable sensor–based fall detection algorithm.

#### 2.2.2. Machine Learning–Based Fall Detection Algorithms

Machine learning–based techniques may overcome the disadvantages of threshold-based techniques. Machine learning is the science of providing the ability of a computer to explore the data construction. Machine learning builds a model using training data to predict or solve the given problem. For fall detection, supervised learning trains the classifier through labeled input data, fall or ADL, during a training period and then identifies individual falls during a classification period [29]. Unsupervised learning algorithms cluster the original data automatically using a clustering algorithm instead of artificially labeling data before training [30]. The accuracy of supervised learning is higher than that of unsupervised learning, but unsupervised learning saves time in the labeling of data. Obviously, high accuracy is needed to fulfill the requirement of a fall detection system. The popular machine learning techniques are naïve Bayesian (NB), support vector machine (SVM), k-nearest neighbors (kNN), radial basis function (RBF), and decision trees. Comparing each machine learning technique in fall detection, Albert et al. [29] performed fall detection by placing a tri-axial accelerometer on the higher part of the subject’s trunk, and the results showed that SVM was able to identify a fall with 98% accuracy, and Özdemir et al. [31] claimed that the kNN classifier gave the best accuracy compared to five classifiers, including the least squares method (LSM), SVM, Bayesian decision-making (BDM), dynamic time warping (DTW), and artificial neural network (ANN). Choi et al. [32] detected falls via two experiments, one with single-node analysis and the other with double-node analysis, which used naïve Bayesian classification to classify the data. The results of the single-node analysis and double-node analysis achieved 99.4% and 99.8% accuracy, respectively.

## 3. Materials and Methods

**Figure 1:**
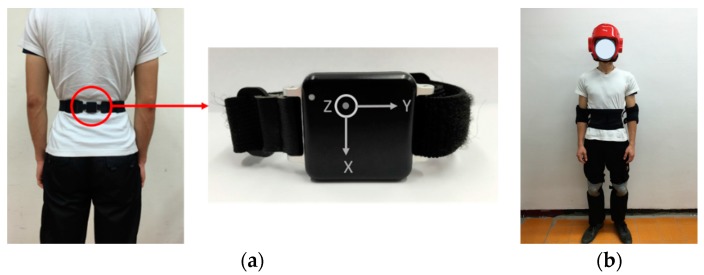
(**a**) Wearing position and the axial direction of sensor; (**b**) Schematic view of the participant wearing a helmet, waist, knee, and elbow guards.

This study used a tri-axial accelerometer that can collect the acceleration generated by body movement, gravitational acceleration, and noise at the same time. The accelerations are generated by a tri-axial accelerometer (OPAL, published by APDM, Portland, USA [33], the range is ±6*g*). Since the duration of the fall event is very short, and to avoid losing important data and/or signal, the fixed sampling rate of the accelerometer is set at 128 Hz. The sensor was attached to the waist, around the lower back, to record the body acceleration. The attached position and definition of coordinate system are shown in Figure 1a. We recruited eight healthy volunteers (males, 22 ± 1.309 years old) into this study. We fitted one sensor to the subject’s waist, and subjects wore a helmet, waist, knee, and elbow guards as show in Figure 1b while performing the falls on a soft mattress to prevent injuries. The subject performed seven types of falls and ADLs, each repeated two or three times in falls and three or five times in ADLs, depending on physical condition of the subject. Based on the previous work [23], the study summarized the common types of falls and ADLs. The types of falls and ADL events which we adopted are presented in Table 2. There are seven kinds of falls, including 25 falling postures, and seven types of ADLs, including 12 postures, that were performed in the experiment. The falls were executed in four falling directions, such as forward, backward, and right- and left-lateral.

### 3.1. Hierarchical Fall Detection Algorithm

**Figure 2:**
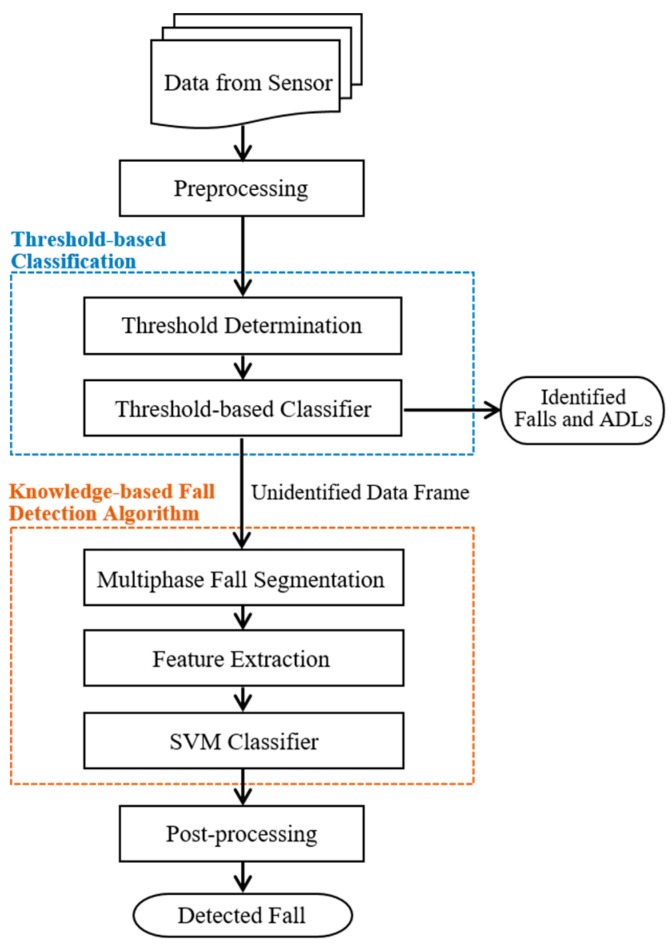
The functional diagram of the proposed fall detection algorithm.

The functional diagram of the proposed hierarchical fall detection algorithm is shown in Figure 2. There are four stages in the hierarchical fall detection algorithm, including preprocessing, threshold-based classification, the knowledge-based fall detection algorithm, and post-processing. In preprocessing, we propose a windowing approach to segment each record with the same window size. Firstly, *Norm_xyz_* is defined as the Euclidean norm of tri-axial accelerations as calculated by Equation (1), where $a_{x}$, $a_{y}$, and $a_{z}$ are the acceleration (*g*) in the *x*-, *y*-, and *z*-axes, respectively. Then the time index corresponding to a maximum of *Norm_xyz_* is identified as the critical point. Finally, the data frame is determined by taking 1.5 s (128 Hz × 1.5 s = 192 samples) before the critical point and 2.5 s (128 Hz × 2.5 s = 320 samples) after the critical point, corresponding to a time window of 513 samples (192 + time index of the critical point + 320).

**Figure 3:**
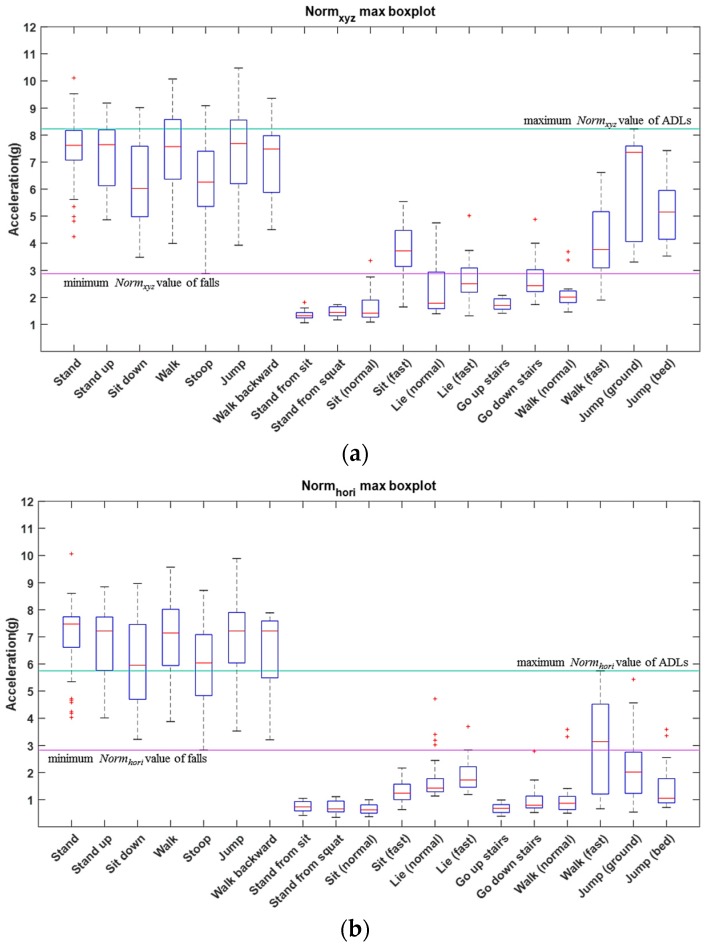
The illustration of the *Norm_xyz_* and *Norm_hori_* distribution of falls and ADLs. The left seven activities are falls and the right 12 activities are ADLs. The green line is determined by the maximum *Norm_xyz_* value of ADLs. The purple line is determined by the minimum *Norm_xyz_* value of falls. (**a**) The boxplot of maximum *Norm_xyz_* values for falls and ADLs; (**b**) The boxplot of maximum *Norm_hori_* values for falls and ADLs.

In this work, the threshold-based classification is utilized to identify the absolute fall and ADL. Two common features can be extracted from each data frame involving *Norm_xyz_* and *Norm_hori_*. *Norm_xyz_* is used to describe the spatial variation of acceleration during the falling interval. *Norm_hori_* is defined as the Euclidean norm of acceleration in the horizontal plane and can be calculated by Equation (2), which is used to describe the change of velocity in the horizontal plane of the body. The threshold is determined by observing the distribution of the maximum *Norm_xyz_* and *Norm_hori_* of the data frames. The *Norm_xyz_* and *Norm_hori_* distribution of falls and ADLs are presented in Figure 3.

(1) $$Norm_{xyz}=\sqrt{a_{x}^{2}+a_{y}^{2}+a_{z}^{2}}$$

(2) $$Norm_{hori}=\sqrt{a_{y}^{2}+a_{z}^{2}}$$

Given that a training set of a data frame $F_{train}=\left\{\left(f_{i}^{train}\right)|i=1,\;2,\ldots ,N_{train}\right\}$, where $N_{train}$ is the total number of the set $F_{train}$, and a testing set of data frame $F_{test}=\left\{\left(f_{i}^{test}\right)|i=1,\;2,\ldots ,N_{test}\right\}$, where $N_{test}$ is the total number of the set $F_{test}$, there are two sets of maximum *Norm_xyz_* and *Norm_hori_* corresponding to $F_{train}$ that can be presented as $V_{train}=\{v_{i}^{train}|i=1,2,\ldots ,N_{train}\}$ and $W_{train}=\{w_{i}^{train}|i=1,\;2,\ldots ,N_{train}\}$, respectively. According to the distribution of $V_{train}$ and $W_{train}$, two thresholds, including $T_{fall}^{Norm_{xyz}}$ and $T_{fall}^{Norm_{hori}}$, for the absolute fall detection can be determined, where $T_{fall}^{Norm_{xyz}}$ and $T_{fall}^{Norm_{hori}}$ are the maximum *Norm_xyz_* and *Norm_hori_* values of ADLs, respectively. Additionally, two thresholds, including $T_{ADL}^{Norm_{xyz}}$ and $T_{ADL}^{Norm_{hori}}$, for the absolute ADL identification can be determined, where $T_{ADL}^{Norm_{xyz}}$ and $T_{ADL}^{Norm_{hori}}$ are the minimum *Norm_xyz_* and *Norm_hori_* values of falls, respectively. Similarly, two sets of maximum *Norm_xyz_* and *Norm_hori_* values corresponding to $F_{test}$ can be presented as $V_{test}=\{v_{i}^{test}|i=1,\;2,\ldots ,N_{test}\}$ and $W_{test}=\left\{w_{i}^{test}|i=1,\;2,\ldots ,N_{test}\right\}$, respectively. Finally, the threshold-based classifier applied to the testing set can be defined as follows:

(3) $$TC\left(v_{i}^{test},\;w_{i}^{test}\right)=\{\begin{array}{c} \mathrm{Fall},\;\mathrm{if}\;v_{i}^{test}>T_{fall}^{Norm_{xyz}}\;\mathrm{and}\;w_{i}^{test}>T_{fall}^{Norm_{hori}}\\ \mathrm{ADL},\;\mathrm{if}\;v_{i}^{test}<T_{ADL}^{Norm_{xyz}}\;\mathrm{and}\;w_{i}^{test}<T_{ADL}^{Norm_{hori}}\\ \mathrm{Unidentified}\;\mathrm{data}\;\mathrm{frame},\;\mathrm{others} \end{array}$$

Fall events can be represented by semantics and rules. A fall is divided into three phases (free fall, impact, and rest phases) based on the attributes of the fall. The free fall phase consists of the sudden movement of the body toward the ground, before hitting the ground, which cannot be rescued by protective strategies. The duration of the free fall phase depends on the subject, and the signal pattern when *Norm_xyz_* is closer to 0*g* has been studied in various durations, including 120–200 ms [22], 300–500 ms [21], and 400–800 ms [43]. The impact phase is determined by the person hitting the ground and has a signal pattern with a sharp pulsation, usually less than one second. In the rest phase, the person remains motionless in a prone or supine position and usually has a mitigating signal pattern. There is potential for the multiphase fall model to be improved, involving important aspects of the fall event. The knowledge-based fall detection algorithm using a multiphase fall model is proposed to provide the system with advanced functionality to deal with unidentified data frames.

**Figure 4:**
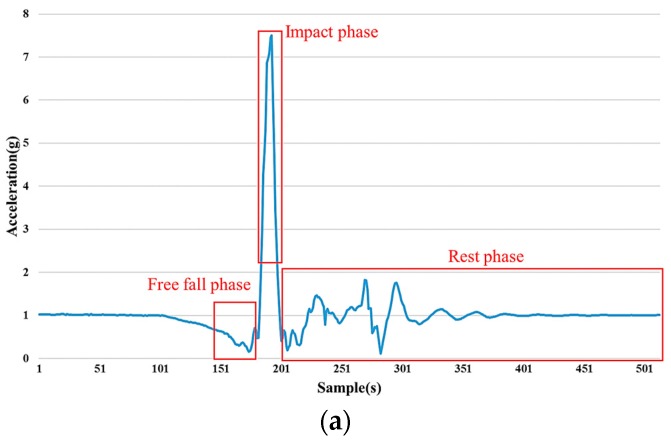

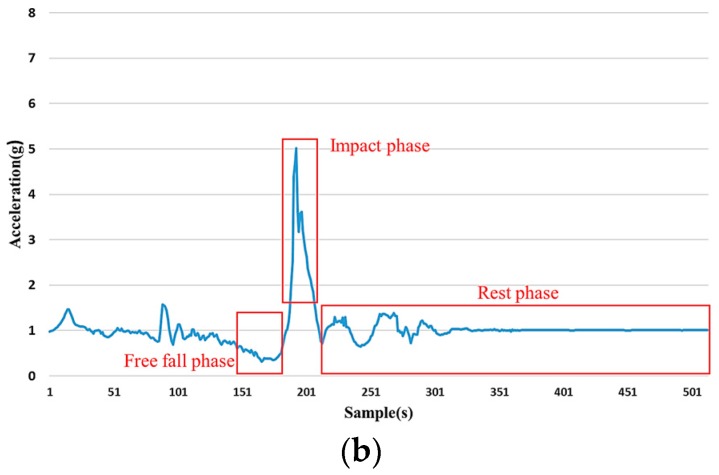
The illustration of the multiphase fall segmentation for two situations. There are three phases, in turn: free fall phase, impact phase, and rest phase. (**a**) The situation of the maximum of *Norm_xyz_* is larger than 6*g*, taking the data frame of the standing forward fall, for example; (**b**) The situation of the maximum of *Norm_xyz_* is less than 6*g*, taking the data frame of the back-walking backward fall for example.

According to observations and the statistical analysis of data frames, the multiphase fall segmentation for the multiphase fall model is presented as follows. To determine the duration of impact phase, two situations are identified based on the severity of falls. One situation is the maximum of *Norm_xyz_* larger than 6*g*, and the other is the maximum of *Norm_xyz_* less than 6*g*. The impact phase of the first situation is determined by taking 10 samples before and after the critical point, and a total of 21 samples (10 + critical point + 10) while the maximum of *Norm_xyz_* is larger than 6*g*. The impact phase of another situation is determined by taking 10 samples before, and 20 samples after, the critical point, for a total of 31 samples (10 + critical point + 20) while the maximum of *Norm_xyz_* is less than 6*g*. Then, the rest phase is determined by taking the following samples after the defined impact phase within the data frame. Finally, the free fall phase is determined by taking 32 samples before the impact phase. The proposed multiphase fall segmentation is sufficient for the knowledge-based fall detection algorithm to identify multiple situations, so that the necessary information can be obtained. The illustration of the multiphase fall segmentation for two situations is shown in Figure 4, and there are three phases, in turn: the free fall phase, impact phase, and rest phase.

Suitable features extracted from signal make for accurate results of the classification model. Based on the previous review study [23], the commonly extracted features were summarized. Time-domain statistical features are selected in this work, including mean, standard deviation, variance, maximum, minimum, range, kurtosis, skewness, and correlation coefficient [44]. The nine features are extracted from each axis including the Euclidean norm of tri-axial acceleration, Euclidean norm of acceleration on the coronal plane, and the Euclidean norm of acceleration on horizontal plane. The list of 54 features used in this work is summarized in Table 3, where m is the number of total samples in a data frame or phase (free fall, impact, and rest phase). All of the features are employed in all of the data frames or phases for training and testing processes.

In the SVM classifier, we use the multi-class SVM technique to train the multiphase fall classifier to classify the fall event into the three distinct phases. SVM is a machine-learning technique originally for binary classification. Training data of the separate categories are divided by a hyperplane and margins that are as wide as possible. A training algorithm trains a classification model, which can classify testing data into one category or the other. Further, binary classification can extend to multi-class classifiers with more than two categories. There are two approaching types for multi-class SVM. One is combining several binary classifiers (one-versus-one technique), and the other is considering all training data in one optimization formulation (one-versus-all technique) [45]. The one-versus-one technique trains x(x−1)/2 binary classifiers for a *x*-class SVM [46]. The outcome’s index of the *x*-class SVM has one positive class and the others have negative classes; that positive class is the predicted result of the *x*-class SVM. The multi-class SVM classifier is applied with the one-versus-one technique and the linear kernel function in this work.

The unidentified data frame in the testing data is divided into three phases following the multiphase fall segmentation. The trained multiphase fall classifier classifies the unidentified phase into a series of phases by feature extraction. In post-processing, we define the temporal order of the fall, listed as free fall, impact, and rest, and check the temporal order from the SVM classifier outcome whether the temporal order is of a fall or not. The fall phase detection and post-processing are defined with the following description: Suppose the acceleration frame $A=\;\left\{a_{1},\;a_{2},a_{3}\right\}$, where $a_{i}=\;\left(s_{i},\;t_{i}\right)$ is the tuple with the fall detection phase $s_{i}$ and timestamp $t_{i}$; then 1≤i≤3. For each fall detection phase $s_{i}\in SF$, and SF is the set of distinct semantic fall phases $SF=\;\left(\left(sf_{j}\right)|1\leq j\leq k\right)$, where k is the number of the defined semantic fall phase. In this work, three semantic fall phases are defined (k = 3) {“FreeFall”, “Impact”, “Rest”}. Finally, we check the tuple of $a_{i}$ in A. The results of post-processing are detected falls when the series classifying free fall, impact, and rest are in order.

### 3.2. Performance Evaluation Criteria

The result of fall detection is either fall or ADL, which belongs to the binary classification. In the performance evaluation criteria of the binary classification test, a positive condition means that the subject falls, and a negative condition means that the subject performed an ADL. Based on the detected result, a fall alarm belongs to the positive test outcome, and an ADL is the result of a negative test outcome. There are four situations in fall detection, including true positive (TP), false positive (FP), true negative (TN), and false negative (FN) [47]. The fall detection system should avoid getting FP and FN results. Sensitivity, specificity, positive predictive value (PPV), negative predictive value (NPV), and accuracy are the common performance evaluation criteria for the binary classification test, and many studies adopted those criteria to show the results of fall detection [24,47,48]. Sensitivity (or recall) is the capability of detecting falls, and the PPV (or precision) is the quality of detecting exact falls. Sensitivity, specificity, precision, and accuracy show more effective evaluation of human activity classification for the imbalanced dataset [49,50]. The sensitivity, specificity, and precision are computed by Equations (4)–(6), respectively. Accuracy is the proportion of the truth test outcome in the total results, whose calculation follows Equation (7). The higher values of sensitivity, specificity, precision, and accuracy, the higher the performance the system provides.

(4) $$Sensitivity=\frac{TP}{TP+FN}$$

(5) $$Specificity=\frac{TN}{FP+TN}$$

(6) $$Precision=\frac{TP}{TP+FP}$$

(7) $$Accuracy=\frac{TP+TN}{TP+FP+TN+FN}$$

## 4. Results and Discussion

The results of the performance evaluation were obtained by the *k*-fold cross-validation method and repeated over five rounds, meaning we chose *k* = 5 for the *k*-fold cross-validation method. The *k*-fold cross-validation method randomly partitions the collected data into *k* portions and utilizes *k* − 1 portions as training data and one portion as testing data, then repeating it k times to evaluate the results. The total number of falls and ADLs were 475 and 364, respectively. Further, the total number of each posture, including stand, stand up, sit down, walk, stoop, jump, walk backward, stand from sit, stand from squat, sit (normal), sit (fast), lie (normal), lie (fast), go upstairs, go downstairs, walk (normal), walk (fast), jump (ground) and jump (bed), was 76, 76, 76, 76, 76, 76, 19, 30, 30, 30, 30, 29, 30, 37, 29, 30, 30, 29, and 30, respectively. The total data and false predictions of falls and ADLs in each round of the five-fold cross-validation are presented in Appendix A in detail. The total testing data of falls and ADLs in each round are shown in Table A1. The testing data of false predictions for falls and ADLs are represented in Table A2 and Table A3.

The threshold-based classification was utilized to identify the absolute falls or ADLs and to decrease the computing complexity in the knowledge-based fall detection algorithm. The identified average percentage of falls and ADLs by the initial threshold-based classification was 12.92% and 27.84%, respectively. Thus, there was an unidentified average percentage of 59.24% in the input data of the knowledge-based fall detection algorithm. In comparison with the proposed knowledge-based fall detection algorithm, the knowledge-based approach without the multiphase fall model, called the machine learning-based fall detection algorithm, was also applied in this work. The average accuracy and standard deviations of each five-fold cross-validation over five rounds for the knowledge-based and machine learning–based fall detection algorithms are presented in Table 4 and Table 5, respectively. The performances of the sensitivity, specificity, precision, and accuracy using knowledge-based algorithms were higher than the performances using machine learning–based methods in each round.

The standard deviations of the performances using the proposed algorithm were lower than those using machine learning–based methods in each round. The overall performances of the sensitivity, specificity, precision, and accuracy using the knowledge-based algorithm were 99.79%, 98.74%, 99.05%, and 99.33%, respectively. Considering the same round, the highest sensitivity and precision were 100% and 98.97% in the first round using the knowledge-based fall detection algorithm, and 99.58% and 98.97% using the machine learning–based fall detection algorithm. Precision is the quality of detecting exact falls and the highest performance of the proposed algorithm was 99.17%. Compared to using the machine learning–based algorithm, the improvement in the overall performances of sensitivity, specificity, precision, and accuracy using the knowledge-based algorithm were 0.55%, 0.33%, 0.24% and 0.45%, respectively.

The confusion matrices of the knowledge-based and machine learning–based algorithms for each type of fall and ADL are shown in Table 6 and Table 7, respectively. Two confusion matrices present the numbers classified by the two algorithms from the total testing data with five rounds. The results show that the false -negative rate using the knowledge-based algorithm in stoop was 1.32%, and the false-negative rates using the machine learning–based algorithm in sit down, stoop, jump, and walk backward were 0.79%, 2.1%, 0.53% and 5.26%, respectively. Obviously, the false predicted types, as well as the numbers of false predicted falls, using the proposed algorithm were less than those when using the machine learning–based algorithm. Similarly, the types of false classified ADLs using the proposed algorithm were less than those using the machine learning–based algorithm. However, the proposed algorithm was still too weak to deal with the ADLs in the lie on the bed activity at normal and fast speeds. This is because the pattern of falls and lying are inherently similar, and the experiment assumed that the faller lost consciousness. Furthermore, the soft mattress set on the ground in the experimental environment caused some subjects to perform the lie on the bed activity at a normal or fast speed, which led to a larger impact signal on the soft mattress and, thus, a similar pattern to a fall. Such falsely predicted ADLs can be improved significantly through the additional information from the context. For example, most lying activities exist when the subject lies on the bed in the bedroom. Additional context information, such as object interaction and location, can provide a fall detection system with the ability to exclude normal ADLs.

Becker et al. [22] proposed a multiphase fall model based on real-world fall recordings. The multiphase fall model contains pre-fall, falling, impact, resting, and recovery phases. We adopted falling, impact, and resting phases as core phases of the fall model in this study, while the experiment assumed that the faller lost consciousness. Fortino et al. [51] proposed a two-stage fall detection system. The first stage is a threshold-based trigger and the second stage utilizes posture recognition of the recovery phase to classify the different levels of alarm severity. For example, the recovery phase may be as important as the impact phase and can judge the severity of a fall event from a clinical perspective. The pre-fall phase can provide more information for the caregiver or healthcare professional when the faller has forgotten or misremembered the circumstances about the fall event. Kangas et al. [52] presented that the impact signal of a fall in an emulation experiment is lower than in real-world falls. The smaller impact signal of a fall may result in ADLs and falls having similar impact signals, which poses a challenging task for the fall detection algorithm. The experimental results have shown that the proposed algorithm can efficiently deal with such challenging tasks and it improves the performance of the machine learning–based algorithm. Furthermore, the meaningful segmentation based on the multiphase fall model can tackle the issue of ambiguity, while some short-duration ADLs have similar attributes to falls, such as sitting down, standing up, and jumping.

The standard deviation of the performances can indicate the stability of the algorithm and/or the system. Compared to a previous study [31], the standard deviation of the performances had a higher score in this work. The standard deviation of the performances was larger than the gain in specificity and precision in this work. Perhaps the greatest reason is that the number of data frames for the testing and training processes was smaller. A larger number of data frames that can train a robust model can provide a more correct classification, thus achieving a more stable algorithm. In future work, we plan to include more emulation experiments for validation, and to consider more phases of a fall to develop a robust fall detection algorithm. Such robust fall detection algorithms have great potential to be successfully applied in various applications involving fall prevention and long-term fall event recording. Further, the real-time automatic fall detection system based on the proposed algorithm will be implemented in the real world.

## 5. Conclusions

The problems generated by fall accidents are important issues in an aging and aged society. Rescuing the victims in time can not only reduce injuries caused by falls but can also increase the confidence in performing ADLs in the elderly. The automatic fall detection system has the opportunity to provide real-time emergency alarms and services for improving safety and health-related quality of life. In this work, we presented a novel hierarchical fall detection algorithm to detect fall events by using a multiphase fall model. Two sensing domains were analyzed, which are directly linked to the sequence of ADLs and fall events. The evaluation results showed that the proposed hierarchical fall detection algorithm can efficiently deal with the issues of the individual differences, such as adaptability and flexibility. Different from previous studies with respect to the machine learning–based method for fall detection, the proposed fall detection algorithm classified the phases—free fall, impact, and rest on the ground—during falling. The overall performances of sensitivity, specificity, precision, and accuracy using the knowledge-based algorithm were 99.79%, 98.74%, 99.05%, and 99.33%, respectively. The improvement of the overall performances of sensitivity, specificity, precision, and accuracy using the knowledge-based algorithm were 0.55%, 0.33%, 0.24%, and 0.45%, respectively, compared to using the machine learning–based algorithm. In future work, we plan to refine the fall detection system for continuous monitoring, and evaluate it in an out-of-lab environment.

## Figures and Tables

**Table 1:** Literature of automatic fall detection algorithms.

Article (Year)	Detection Algorithm (Methods)	Sensor(s)	Placement	Features Used for Fall Detection	Fall and ADL Types	Results
Kangas et al. (2008) [28]	Threshold-based	Tri-axial accelerometer	Waist Wrist Head	Beginning of the fall (SV_TOT_) Falling velocity Fall impact (SV_TOT_, SV_D_, SV_MaxMin_, or Z_2_) Posture after impact	Falls: 9 ADLs: --	Sn ^2^: 97% (Waist) Sp ^2^: 100% (Waist)
Dinh et al. (2009) [34]	Machine learning–based (NB, RBF, SVM, C4.5 Ripple down rule learner)	Tri-axial accelerometer Dual-axial gyroscope	Thorax	Acceleration (*X*, *Y* and *Z* axis) Gyroscope (*X* and *Y* axis)	Falls: 4 ADLs: 3	Naïve Bayesian Acc ^2^: 97.3% Radial Basis Function Acc ^2^: 95.8%
Chao et al. (2009) [35]	Threshold-based	Tri-axial accelerometer	Chest Waist	Acceleration magnitude Acceleration cross-product	Falls: 8 ADLs: 13	Sn ^2^: 98.2% (Chest) Sp ^2^: 92.4% (Chest) Sn ^2^: 98.2% (Waist) Sp ^2^: 89.9% (Waist)
Bourke et al. (2010) [36]	Threshold-based	Tri-axial accelerometer	Waist	Upper fall threshold Lower fall threshold Vertical velocity	Falls: 8 ADLs: 4	Velocity + impact + posture Sn ^2^: 100% Sp ^2^: 100% Less than 1 false positive a day
Choi et al. (2011) [32]	Machine learning–based (NB)	SNA ^1^: Tri-axial accelerometer, dual-axial gyroscope DNA ^1^: Tri-axial accelerometer, one-axial gyroscope	SNA ^1^: Chest DNA ^1^: Chest, Thigh	SNA ^1^: Acceleration (*X*, *Y* and *Z* axis) Gyroscope (*X* and *Y* axis) DNA ^1^: Acceleration (*X*, *Y* and *Z* axis) Gyroscope (*X* axis)	SNA ^1^: Falls: 4 ADLs: 3 DNA ^1^: Falls: 4 ADLs: 4	SNA ^1^: Acc ^2^: 99.4% DNA ^1^: Acc ^2^: 99.8%
Rescio et al. (2013) [37]	Machine learning–based (SVM)	Tri-axial accelerometer	Waist	The product between the value of the acceleration peak and the change in the CPO	--	Sn ^2^: 97.7% Sp ^2^: 94.8%
Özdemir et al. (2014) [31]	Machine learning–based (kNN, LSM, SVM, Bayesian Decision Making, Dynamic Time Warping, ANN)	Tri-axial accelerometer Tri-axial gyroscope Tri-axial magnetometer	Head, Chest, Waist, Wrist, Thigh, Ankle	Minimum, Maximum Mean, Variance Skewness Kurtosis Autocorrelation Discrete Fourier transform	Falls: 20 ADLs: 16	kNN Sn ^2^: 100% Sp ^2^: 99.91%
Huynh et al. (2015) [38]	Threshold-based	Tri-axial accelerometer Tri-axial gyroscope	Chest	Upper fall threshold Lower fall threshold	Falls: 4 ADLs: 6	Sn ^2^: 96.55% Sp ^2^: 89.50%
Palmerini et al. (2015) [39]	Threshold-based	Tri-axial accelerometer	Lower back	Continuous wavelet transform coefficients Upper peak value Lower peak value	Falls: 5 ADLs: --	Wavelet Sn ^2^: 90% Sp ^2^: 89.7%
He et al. (2016) [40]	Machine learning–based (kNN, NB, Bayes Net, ANN, Decision Tree, Bagging, Ripper)	Tri-axial accelerometer Tri-axial gyroscope	upper trunk	Resultant acceleration (α) Resultant angular velocity (ω)	Falls: 2 ADLs: 3	kNN (*k* = 3) Sn ^2^: 100% Sp ^2^: 99.91% Acc ^2^: 97.8548%
Chen et al. (2016) [41]	Machine learning–based (SVM)	Tri-axial accelerometer	Waist	Maximum magnitude of the sum vector Rotation angle Slope Acceleration in the xy-plane Standard deviation of the sun vector	Falls: 6 ADLs: 6	Sn ^2^: 95.76% Sp ^2^: 93.28% Acc ^2^: 94.58%
Gibson et al. (2016) [42]	Machine learning-based (ANN, kNN, RBF, Probabilistic Principal Component Analysis, Linear Discriminant Analysis)	Tri-axial accelerometer	Chest	Discrete wavelet transform	Falls: 6 ADLs: 5	Radial Basis Function Sn ^2^: 100% Sp ^2^: 99.91% Linear Discriminant Analysis Sn ^2^: 100% Sp ^2^: 99.91%

^1^ SNA: single-node analysis; DNA: double-node analysis; ^2^ Sn: sensitivity; Sp: specificity; and Acc: accuracy.

**Table 2:** The types and characteristics of falls and ADLs for experiments.

**No.**	**Activities before Fall**	**Characteristics**			
1	Stand	Forward		Backward	Lateral (right and left)
2	Stand up	Forward		Backward	Lateral (right and left)
3	Sit down	Forward		Backward	Lateral (right and left)
4	Stoop	Forward		Backward	Lateral (right and left)
5	Walk	Forward		Backward	Lateral (right and left)
6	Walk backward	--		Backward	--
7	Jump	Forward		Backward	Lateral (right and left)
**No.**	**Activities of Daily Living**	**Characteristic**	**No.**	**Activities of Daily Living**	**Characteristic**
1	Stand up	From sit	2	Stand up	From squat
3	Sit down	Normal	4	Sit down	Fast
5	Lie on the bed	Normal	6	Lie on the bed	Fast
7	Go up stairs	Normal	8	Go down stairs	Normal
9	Walk	Normal	10	Walk	Fast
11	Jump	On the ground	12	Jump	On the bed

**Table 3:** The feature vector for the knowledge-based fall detection algorithm.

Feature Vector, F = (f_1_, f_2_, …, f_54_) ∈ R_54_	Feature Description
f_1_~f_3_	mean *a_x_*(*i*); mean *a_y_*(*i*); mean *a_x_*(*i*), where *i* = 1, ..., *m* ^1^
f_4_~f_6_	mean *a_norm_*(*i*) ^2^; mean *a_verti_*(*i*) ^3^; mean *a_hori_*(*i*) ^4^, where *i* = 1, ..., *m* ^1^
f_7_~f_9_	std *a_x_*(*i*); std *a_y_*(*i*); std *a_x_*(*i*), where *i* = 1, ..., m ^1^
f_10_~f_12_	std *a_norm_*(*i*) ^2^; std *a_verti_*(*i*) ^3^; std *a_hori_*(*i*) ^4^, where *i* = 1, ..., *m* ^1^
f_13_~f_15_	var *a_x_*(*i*); var *a_y_*(*i*); var *a_x_*(*i*), where *i* = 1, ..., m ^1^
f_16_~f_18_	var *a_norm_*(*i*) ^2^; var *a_verti_*(*i*) ^3^; var *a_hori_*(*i*) ^4^, where *i* = 1, ..., *m* ^1^
f_19_~f_21_	max *a_x_*(*i*); max *a_y_*(*i*); max *a_x_*(*i*), where *i* = 1, ..., m ^1^
f_22_~f_24_	max *a_norm_*(*i*) ^2^; max *a_verti_*(*i*) ^3^; max *a_hori_*(*i*) ^4^, where *i* = 1, ..., *m* ^1^
f_25_~f_27_	min *a_x_*(*i*); min *a_y_*(*i*); min *a_x_*(*i*), where *i* = 1, ..., m ^1^
f_28_~f_30_	min *a_norm_*(*i*) ^2^; min *a_verti_*(*i*) ^3^; min *a_hori_*(*i*) ^4^, where *i* = 1, ..., *m* ^1^
f_31_~f_33_	range *a_x_*(*i*); range *a_y_*(*i*); range *a_x_*(*i*), where *i* = 1, ..., *m* ^1^
f_34_~f_36_	range *a_norm_*(*i*) ^2^; range *a_verti_*(*i*) ^3^; range *a_hori_*(*i*) ^4^, where *i* = 1, ..., *m* ^1^
f_37_~f_39_	kurtosis *a_x_*(*i*); kurtosis *a_y_*(*i*); kurtosis *a_x_*(*i*), where *i* = 1, ..., *m* ^1^
f_40_~f_42_	kurtosis *a_norm_*(*i*) ^2^; kurtosis *a_verti_*(*i*) ^3^; kurtosis *a_hori_*(*i*) ^4^, where *i* = 1, ..., *m* ^1^
f_43_~f_45_	skewness *a_x_*(*i*); skewness *a_y_*(*i*); skewness *a_x_*(*i*), where *i* = 1, ..., *m* ^1^
f_46_~f_48_	skewness *a_norm_*(*i*) ^2^; skewness *a_verti_*(*i*) ^3^; skewness *a_hori_*(*i*) ^4^, where *i* = 1, ..., *m* ^1^
f_49_	Correlation coefficient between *a_x_* and *a_y_*
f_50_	Correlation coefficient between *a_x_* and *a_z_*
f_51_	Correlation coefficient between *a_y_* and *a_z_*
f_52_	Correlation coefficient between *a_norm_* ^2^ and *a_verti_* ^3^
f_53_	Correlation coefficient between *a_norm_* ^2^ and *a_hori_* ^4^
f_54_	Correlation coefficient between *a_verti_* ^3^ and *a_hori_* ^4^

^1^ *m*: Determined size of data frame (or phase); ^2^ *a_norm_*: Euclidean norm of tri-axial acceleration; ^3^ *a_verti_*: Euclidean norm of acceleration on coronal plane; ^4^ *a_hori_*: Euclidean norm of acceleration on horizontal plane.

**Table 4:** The overall performance of five-fold cross-validation over five rounds for the knowledge-based fall detection algorithm. Std: standard deviation.

Knowledge-Based Fall Detection Algorithm						
Round	1	2	3	4	5	Mean (Std)
Sensitivity (%)	100 (0)	99.79 (0.47)	99.58 (0.57)	99.79 (0.48)	99.79 (0.47)	99.79 (0.43)
Specificity (%)	98.63 (1.36)	98.62 (1.00)	98.90 (0.61)	98.62 (0.98)	98.91 (0.61)	98.74 (0.88)
Precision (%)	98.97 (1.03)	98.96 (0.73)	99.16 (0.47)	98.97 (0.72)	99.17 (0.47)	99.05 (0.66)
Accuracy (%)	99.41 (0.59)	99.29 (0.50)	99.29 (0.26)	99.28 (0.27)	99.41 (0.42)	99.33 (0.40)

**Table 5:** The overall performance of five-fold cross-validation over five rounds for the machine learning–based fall detection algorithm.

Machine Learning–Based Fall Detection Algorithm						
Round	1	2	3	4	5	Mean (Std)
Sensitivity (%)	99.58 (0.58)	99.16 (1.37)	99.36 (0.94)	99.15 (0.89)	98.95 (0.74)	99.24 (0.89)
Specificity (%)	98.63 (1.68)	98.63 (1.66)	98.63 (2.37)	97.80 (2.50)	98.37 (1.48)	98.41 (1.84)
Precision (%)	98.97 (1.26)	98.95 (1.29)	98.99 (1.73)	98.35 (1.88)	98.76 (1.10)	98.81 (1.38)
Accuracy (%)	99.17 (0.90)	98.93 (1.47)	99.05 (0.90)	98.56 (1.02)	98.69 (0.49)	98.88 (0.95)

**Table 6:** The confusion matrix of the knowledge-based algorithm for each fall and ADL. The number of falls and ADLs is the total number of five-fold cross-validations over five rounds.

Predict Results and Measure Matrix	Fall (Ground Truth)							ADL (Ground Truth)											
	Stand	stand up	Sit down	Walk	Stoop	Jump	Walk Backward	Stand from Sit	Stand from Squat	Sit (Normal)	Sit (Fast)	Lie (Normal)	Lie (Fast)	Go up Stairs	Go down Stairs	Walk (Normal)	Walk (Fast)	Jump (Ground)	Jump (Bed)
Fall (Predicted)	380	380	380	380	375	380	95	0	0	0	0	8	15	0	0	0	0	0	0
ADL (Predicted)	0	0	0	0	5	0	0	150	150	150	150	137	135	185	145	150	150	145	150
Sensitivity (%)	100	100	100	100	98.68	100	100	--	--	--	--	--	--	--	--	--	--	--	--
Specificity (%)	--	--	--	--	--	--	--	100	100	100	100	94.48	90	100	100	100	100	100	100
False positive rate (%)	--	--	--	--	--	--	--	0	0	0	0	5.52	10	0	0	0	0	0	0
False negative rate (%)	0	0	0	0	1.32	0	0	--	--	--	--	--	--	--	--	--	--	--	--

**Table 7:** The confusion matrix of the machine learning–based algorithm for each fall and ADL. The number of falls and ADLs is the total number of five-fold cross-validations over five rounds.

Predict Results and Measure Matrix	Fall (Ground Truth)							ADL (Ground Truth)											
	Stand	Stand up	Sit down	Walk	Stoop	Jump	Walk Backward	Stand from Sit	Stand from Squat	Sit (Normal)	Sit (Fast)	Lie (Normal)	Lie (Fast)	Go up Stairs	Go down Stairs	Walk (Normal)	Walk (Fast)	Jump (Ground)	Jump (Bed)
Fall (Predicted)	380	380	377	380	372	378	90	0	0	0	4	8	11	0	0	1	4	1	0
ADL (Predicted)	0	0	3	0	8	2	5	150	150	150	146	137	139	185	145	149	146	144	150
Sensitivity (%)	100	100	99.21	100	97.76	99.47	94.74	--	--	--	--	--	--	--	--	--	--	--	--
Specificity (%)	--	--	--	--	--	--	--	100	100	100	97.33	94.48	91.86	100	100	99.33	97.33	99.31	100
False positive rate (%)	--	--	--	--	--	--	--	0	0	0	2.67	5.52	8.14	0	0	0.67	2.67	0.69	0
False negative rate (%)	0	0	0.79	0	2.1	0.53	5.26	--	--	--	--	--	--	--	--	--	--	--	--

## Appendix A

**Table A1:** The total testing data of falls and ADLs in each round of five-fold cross-validation rounds.

Round	Stand	Stand up	Sit down	Walk	Stoop	Jump	Walk Backward	Stand from Sit	Stand from Squat	Sit (Normal)	Sit (Fast)	Lie (Normal)	Lie (Fast)	Go up Stairs	Go down Stairs	Walk (Normal)	Walk (Fast)	Jump (Ground)	Jump (Bed)
1	15	15	16	16	15	15	3	6	6	6	6	6	6	7	6	6	6	6	6
	16	15	15	15	15	15	4	6	6	6	6	6	6	8	6	6	6	6	6
	15	15	15	15	16	16	4	6	6	6	6	6	6	7	6	6	6	5	6
	15	16	15	15	15	15	4	6	6	6	6	6	6	7	6	6	6	6	6
	15	15	15	15	15	15	4	6	6	6	6	5	6	8	5	6	6	6	6
2	15	15	15	16	15	15	4	6	6	6	6	5	6	7	5	6	6	6	6
	16	15	15	15	15	16	4	6	6	6	6	6	6	7	6	6	6	5	6
	15	16	16	15	15	15	4	6	6	6	6	6	6	8	6	6	6	6	6
	15	15	15	15	16	15	4	6	6	6	6	6	6	8	6	6	6	6	6
	15	15	15	15	15	15	3	6	6	6	6	6	6	7	6	6	6	6	6
3	15	15	15	15	15	15	3	6	6	6	6	6	6	7	6	6	6	5	6
	15	15	15	16	15	15	4	6	6	6	6	6	6	7	6	6	6	6	6
	15	15	15	15	15	16	4	6	6	6	6	6	6	7	6	6	6	6	6
	15	16	15	15	16	15	4	6	6	6	6	5	6	8	6	6	6	6	6
	16	15	16	15	15	15	4	6	6	6	6	6	6	8	5	6	6	6	6
4	16	15	16	15	15	15	4	6	6	6	6	6	6	8	6	6	6	6	6
	15	16	15	16	15	15	4	6	6	6	6	6	6	7	6	6	6	6	6
	15	15	15	15	15	15	3	6	6	6	6	5	6	8	5	6	6	6	6
	15	15	15	15	15	15	4	6	6	6	6	6	6	7	6	6	6	6	6
	15	15	15	15	16	16	4	6	6	6	6	6	6	7	6	6	6	5	6
5	16	15	15	16	15	15	4	6	6	6	6	6	6	7	6	6	6	6	6
	15	15	16	15	16	15	4	6	6	6	6	6	6	7	5	6	6	6	6
	15	15	15	15	15	15	4	6	6	6	6	5	6	7	6	6	6	5	6
	15	16	15	15	15	16	4	6	6	6	6	6	6	8	6	6	6	6	6
	15	15	15	15	15	15	3	6	6	6	6	6	6	8	6	6	6	6	6
total	380	380	380	380	380	380	95	150	150	150	150	145	150	185	145	150	150	145	150

**Table A2:** The total testing data of false predictions for falls and ADLs using the knowledge-based algorithm.

Round	Stand	Stand up	Sit down	Walk	Stoop	Jump	Walk Backward	Stand from Sit	Stand from Squat	Sit (Normal)	Sit (Fast)	Lie (Normal)	Lie (Fast)	Go up Stairs	Go down Stairs	Walk (Normal)	Walk (Fast)	Jump (Ground)	Jump (Bed)
1												1	1						
												1	1						
													1						
																		
																		
2												2							
													1						
													1						
					1								1						
																		
3													1						
													1						
					1														
												1							
					1								1						
4													1						
												1							
												1							
					1														
													2						
5					1								1						
												1							
																		
													1						
													1						
total	0	0	0	0	5	0	0	0	0	0	0	8	15	0	0	0	0	0	0

**Table A3:** The total testing data of false predictions for falls and ADLs using the machine learning–based algorithm.

Round	Stand	Stand up	Sit down	Walk	Stoop	Jump	Walk Backward	Stand from Sit	Stand from Squat	Sit (Normal)	Sit (Fast)	Lie (Normal)	Lie (Fast)	Go up Stairs	Go down Stairs	Walk (Normal)	Walk (Fast)	Jump (Ground)	Jump (Bed)
1			1									2	1						
													1						
													1						
							1												
																		
2												1							
							1									1			
																		
			1		2						3								
																		
3							1												
													1						
					2														
																		
												2	2						
4												1	2						
							1												
						1					1	1					1	1	
					2														
													1						
5					1	1							1						
							1					1							
			1																
																	3		
					1								1						
total	0	0	3	0	8	2	5	0	0	0	4	8	11	0	0	1	4	1	
